# Tunable Picosecond Laser Pulses via the Contrast of Two Reverse Saturable Absorption Phases in a Waveguide Platform

**DOI:** 10.1038/srep26176

**Published:** 2016-05-18

**Authors:** Yang Tan, Lianwei Chen, Dong Wang, Yanxue Chen, Shavkat Akhmadaliev, Shengqiang Zhou, Minghui Hong, Feng Chen

**Affiliations:** 1 School of Physics, State Key Laboratory of Crystal Materials, Shandong University, Jinan, 250100, China; 2 NUS Graduate School for Integrative Sciences and Engineering, National University of Singapore, 28 Medical Drive, 117456, Singapore; 3 Helmholtz-Zentrum Dresden-Rossendorf, Institute of Ion Beam Physics and Materials Research, Dresden, 01314, Germany; 4 Department of Electrical and Computer Engineering, National University of Singapore, 4 Engineering Drive 3, 117576, Singapore

## Abstract

How to enhance the optical nonlinearity of saturable absorption materials is an important question to improve the functionality of various applications ranging from the high power laser to photonic computational devices. We demonstrate the saturable absorption (SA) of VO_2_ film attributed to the large difference of optical nonlinearities between the two states of the phase-transition materials (VO_2_). Such VO_2_ film demonstrated significantly improved performance with saturation intensity higher than other existing ultrathin saturable absorbers by 3 orders due to its unique nonlinear optical mechanisms in the ultrafast phase change process. Owing to this feature, a Q-switched pulsed laser was fabricated in a waveguide platform, which is the first time to achieve picosecond pulse duration and maintain high peak power. Furthermore, the emission of this VO_2_ waveguide laser can be flexibly switched between the continuous-wave (CW) and pulsed operation regimes by tuning the temperature of the VO_2_ film, which enables VO_2_-based miniature laser devices with unique and versatile functions.

The progress in the nonlinear optical material research has paved the way for many revolutionary inventions, such as the photonic computational devices, high power lasers, and super-resolution optical imaging techniques^1^,^2^,^3^,^4^,^5^. Intensive researches have been conducted to improve the optical nonlinearity of the materials, which are crucial for the functionality, energy consumption, accuracy and working conditions of those devices^6^,^7^,^8^.

There is a continuous requirement for the improvement of the nonlinear materials, in order to satisfy the ever growing need of the advanced photonic devices. For instance, how to produce the pulsed laser with high peak power is an important question for super-resolution bio-imaging microscopy and advanced materials processing^9^,^10^,^11^. Among all the methods, the passive Q-switching based on saturable absorption is more promising to produce such high power output^12^,^13^,^14^,^15^,^16^. However, the intra-cavity intensity of the waveguide laser is commonly lower than several megawatts per square centimeter (MW/cm^2^), which is below the saturation intensity of most saturable absorbers. As a result, it is difficult to obtain the Q-switched waveguide laser in the picosecond timescale^17^,^18^,^19^.

The conventional saturable absorption mechanisms are based on the photon-induced electron excitation. In this nonlinear process, the electrons are further excited from the first excited state to higher energy states. As the life time of the excited electrons is very short, large light intensity is required to maintain the population of excited electrons. Therefore it is fundamentally difficult to greatly reduce the saturation intensity^20^,^21^,^22^.

In this work, the saturable absorption relied on a different mechanism, i.e., Contrast Nonlinear Transmission, has been proved to have more optimal nonlinear behaviors. It is based on the ultrafast phase transition of the VO_2_ to manipulate the transmittance of the light. Such light-induced ultrafast phase transition happens within 300 fs, which enables VO_2_ a unique material among other types of phase transition ones^23^. Previous studies mainly focused on the optical properties in these two phases separately^23^,^24^,^25^. Although VO_2_ was found to be reverse saturable absorbers in both the insulating and metallic phase, this material behaves like saturable absorber in the phase transition process^26^,^27^. In 1995, the saturable absorption of the VO_2_ was firstly discussed^26^. In this work, based on the Contrast Nonlinear Transmission, a significantly improved design, i.e., a tunable pulsed waveguide laser with picosecond duration, was proposed and implemented based on passive Q-switching. Output power up to 200 mW has been achieved in this novel solid-state waveguide laser system. In addition, the temperature tuning of the VO_2_ film has been applied to develop unique CW-pulsed switching laser system.

## Results and Discussion

### Fabrication of VO_2_ film

**Figure 1:**
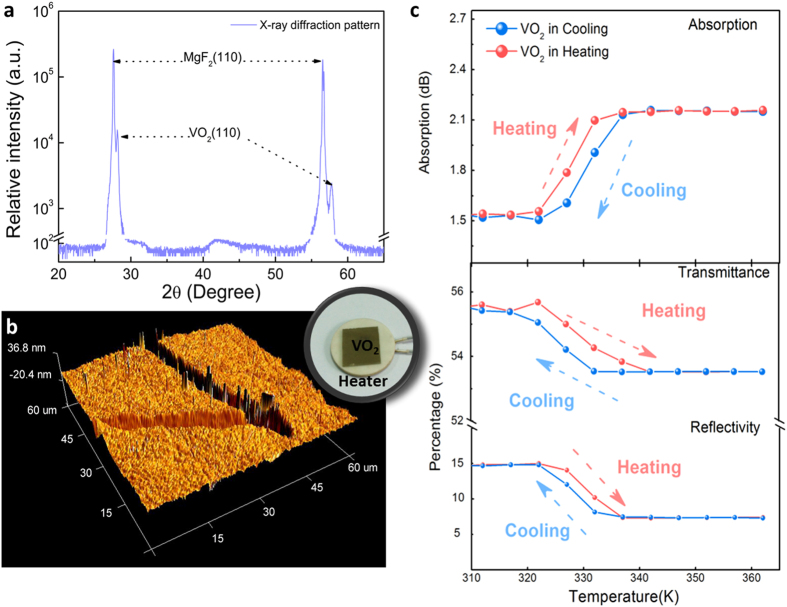
(**a**) X-ray diffraction pattern of the VO_2_ film on MgF_2_ substrate. (**b**) AFM image of the VO_2_ surface, top-right inset: VO_2_ film optical image. (**c**) Linear optical properties at different temperatures (transmittance, reflectivity, absorption).

The ultrathin VO_2_ film was fabricated by the Pulsed Laser Deposition (PLD). An MgF_2_ wafer with the dimension of 10 × 10 × 0.5 mm^3^ was used as the substrate. Figure 1a shows the X-ray diffraction (XRD) plot of the sample. The peaks at the 28.0° and 57.7° correspond to the monoclinic VO_2_ crystal in its mono-crystalline form^26^. The morphology of the VO_2_ film was characterized by an Atomic Force Microscope (Fig. 1b, scratches are made to measure the thickness of the film). The thickness of the film was measured to be ~50 nm. It can be seen from the figure that the VO_2_ surface is relatively smooth and the roughness root mean square (RMS) is calculated to be 2.2 nm, describing an integral characteristic feature of a smooth surface. The electric properties of the sample were measured at different temperatures (Data in supplementary materials S1). The thermal hysteresis effect from 300 to 360 K can be observed in the characterization process. All these data are coherent with the published literatures, which confirm that the VO_2_ film has been well deposited on the MgF_2_ substrate^28^,^29^,^30^,^31^.

### Optical properties of the VO_2_ film

#### Linear Optical Properties

The linear optical properties of the VO_2_ film were characterized at the wavelength of 1064 nm to collect the background information for the nonlinear characterizations. The measurements of the reflectivity (*R*) and transmittance (*T*) were conducted from 320 to 360 K and the absorption (*α*_L_) coefficients were also calculated. It can be seen from Fig. 1c that the obvious drop of the reflectivity occurred when the sample was heated up from ~328 K (similar reflectivity variation can be observed in the cooling process), which indicates the transition of the crystal structure from the monoclinic phase (insulating) to the rutile phase (metallic). The transmittance also decreases with the temperature. However, the change of the linear transmittance is relatively small over the entire phase change process (average 0.067%/K). It can be concluded from this measurement that the phase transition point is at ~330K in which the reflectivity curve decreases the most rapidly. These results are consistent with the previous electric property measurement, in which the phase transition between the insulating and metallic phase also happened at ~330 K. The observed nonlinear phenomenon is related to the series of processes occurring during the photo-induced phase transition of the VO_2_ that was studied over the past fifteen years with ultrafast electron diffraction^32^, transient optical probing^33^, coherent phonon spectroscopy, and THz spectroscopy.

#### Nonlinear Optical Properties

The transition of VO_2_ from the attenuator regime to a saturable absorber is, in the experiments presented in this work, induced by the light^34^ whose intensity reaches the phase transition level. This process is therefore highly out of equilibrium. However, the physics of this transition is probably more complex and the photo-excitation of VO_2_ above a threshold fluence leads to charge distribution that will produce Coulomb interaction^35^ resulting in a lattice potential change^36^ and the Insulator-Metal transition followed by a crystallographic phase transition on a longer time. It is clear that a quantitative understanding of the reported data requires taking into account a more detailed dynamics of the transition processes. However, this nonlinear optical process is difficult to be directly measured in the conventional characterization methods. Instead, the properties of the two phases in their equilibrium conditions are considered to characterize the non-equilibrium contrast transition.

Based on the phase transition information collected in the linear optical property measurement. The third-order nonlinearity characterizations were performed using the *Z*-scan for both two solid phases of the VO_2_ sample. To avoid the disturbance from the accumulated thermal effect, the picosecond laser (1064 nm, 22 ps, pulse energy 0.5 μJ, laser intensity at the focus) was selected to be the light source. It should be noted that even at the focus point (Z = 0 mm), the power is below the threshold for the VO_2_ phase transition^32^. The sample is tuned to its insulating and metallic phase separately by controlling the temperature (293 K for insulating phase and 363 K for metallic phase). Hence the nonlinearity coefficients for the two different phases can be calculated from the measurements.

**Figure 2:**
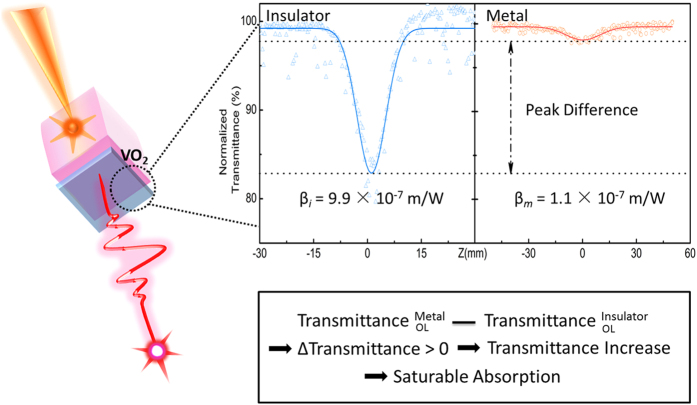
Z-scan results for the VO_2_ film in its insulating phase and metallic phase. Equations: Transmittance difference indicates saturable absorption.

The nonlinear absorption coefficients (*β*) were calculated from the data presented in Fig. 2. The downwards peak in the plot shows the reverse saturable absorption features of the sample. Based on the normalized transmittance in Fig. 2, *β* can be calculated by fitting the following equation with the data^33^:


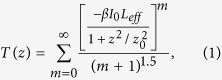


where *z* is the distance of the sample from the focus, *I*_0_ the on-axis peak intensity at the focus, *L*_eff_ the effective interaction length, and *z*_0_ the Rayleigh diffraction length.

The nonlinear absorption coefficient for insulating and metallic phases are calculated to be *β*_*i*_ = 9.9 × 10^−7 ^m/W and *β*_*m*_ = 1.1 × 10^−7 ^m/W, respectively. This difference is due to the different structures in the insulating and the metallic phases^37^,^38^. It can be seen that there is a large variation between the nonlinear behaviors in the two phases. The nonlinear absorption coefficient in insulating phase is 900% higher that of the metallic phase.

#### Q-switched Laser Properties

**Figure 3:**
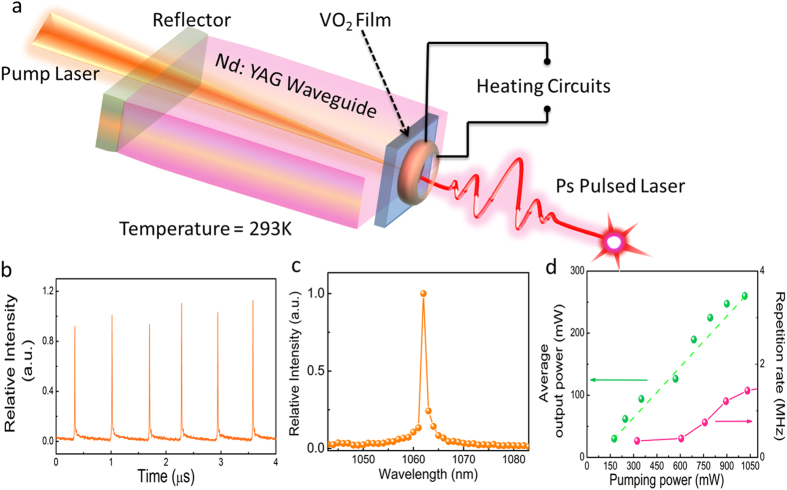
(**a**) Schematic diagram for the Q-switching experimental setup. (**b**) Laser pulse trains at 293 K. (**c**) the relative laser intensity at different wavelengths. (**d**) Average output power and repetition rate at different pumping powers.

To further explore the nonlinear optical property of the VO_2_ film, it was attached to one side of a Nd:YAG waveguide as the output mirror. The nonlinear performance of the VO_2_ film was investigated by the detection of the output waveguide laser. The schematic diagram of the experimental setup is shown in Fig. 3a. Under the cw laser pumping at 810 nm, the pulsed laser emission at 1064 nm was obtained (inset of Fig. 3b) and the pulse train properties are displayed in Fig. 3c,d. It is clear that the pulsed laser was generated with lower repetition rate and higher peak energy compared with the mode-locking waveguide laser^12^,^13^. Besides there was no broadening in the optical spectrum of the output laser. The above results proved that the pulsed laser was generated by the Q-switching mechanism instead of the mode-locking.

In this case, the VO_2_ film served as the saturable absorber. Although the VO_2_ sample demonstrated the reverse saturable absorption features in both the insulating and the metallic phases. The results of this experiment show the contradictory effect of the saturable absorption, which indicate that the intracavity light intensity exceeds the threshold value for the phase transition of VO_2_.

Furthermore, the output pulsed laser signal also presents good features (Fig. 3c). The pulse duration of one single peak is around 700 ps and the conversion slope efficiency of ~23%. Comparing to other similar types of materials which have outputs in nanosecond region, this setup is the first one that can achieve the picosecond pulse duration output in passively Q-switched waveguide laser^17^,^18^,^19^. The short pulse duration means that the laser energy can be concentrated to generate high peak power. The energy can be transferred to the substrate more rapidly and the thermal side effect can be greatly reduced, which is highly demanded for the pulsed laser applications including the non-invasive super-resolution imaging on vulnerable bio-samples and high-precision laser patterning and cutting in semiconductor industry^3^,^10^,^11^.

#### Discussion on Non-equilibrium Contrast Transition

This nonlinear optical process distinguishes itself from the conventional saturable absorption phenomena in a way that the change of the transmittance is not attributed to higher energy states being saturated in a large incident light intensity. Instead, the cause for the optical nonlinearity is the large nonlinear absorption coefficients’ difference and the transition between the two phases.

**Figure 4:**
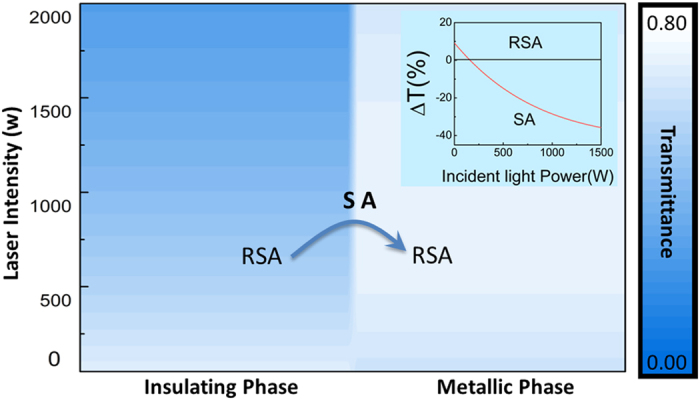
Theoretical calculation for the transmittances of the two phases at different laser intensities. Inset: Contrast of the transmittances of the insulating phase and metallic phase.

It is worth to mention that, due to the phase-transition dynamic nature, Contrast Nonlinear Transmission is very challenging to be directly characterized in the conventional nonlinear optical measurements such as, the *Z*-scan or Input-Output power characterization, which are designed for the steady state materials. We propose a model to characterize the Contrast Nonlinear Transmission based on the nonlinear optical properties in two equilibrium phases, which may be helpful to understand the dynamic progress of the phenomenon. According to the nonlinear absorption coefficients, the linear absorption coefficients, and the saturation intensity determined in the previous experiments, the nonlinear transmittances in different laser intensities were calculated for both the monoclinic phase and the rutile phase. The results are shown in Fig. 4. (the inset shows the transmittance difference of the two phases. Details of the calculation can be found in the supplementary materials S2). As can be seen in the figure, there is an obvious contrast of the transmittances between the two phases. The difference of the nonlinear transmittances are also calculated and shown in the inset. (It is worth to mention that the initial Reverse saturable absorption comes from the difference of the linear absorption coefficients. Details can be found in the supplementary S2). This calculation shows that when the light power exceeds 158W, the VO_2_ demonstrates saturable absorption features in the phase transition process, which is coherent with our experimental data. Another feature is that the Contrast Nonlinear Transmission nonlinearity keeps increasing with the laser intensity and it can be more than 700% larger than the value measured in our experimental condition. However, the gradient of the curve decreases along with the laser intensity, which means that the increase of nonlinearity tends to “saturate” at the very high laser intensity. This finding shows the potentials of the Contrast Nonlinear Transmission systems for the new research direction to explore novel materials and hybrid systems with “super optical nonlinearity”. This can be possibly achieved by further improving the Contrast Nonlinear Transmission systems to possess the transition from the reverse saturable absorption to the saturable absorption and the contrast shall be even larger. Such materials with low saturation intensity and fs response time can be made into photonic logic gates based on nonlinear transmission and lead the realization of all-optical ultrafast photonic computational devices. In industry, nonlinear photonic devices, including the pulsed laser can also utilize this ultrathin, high performance nonlinear material to fabricate its functional components. It is possible to further modify the VO_2_ properties, such as the phase transition temperature, which makes it versatile and tunable to meet other requirements.

#### Thermal effect on Contrast Nonlinear Transmission

**Figure 5:**
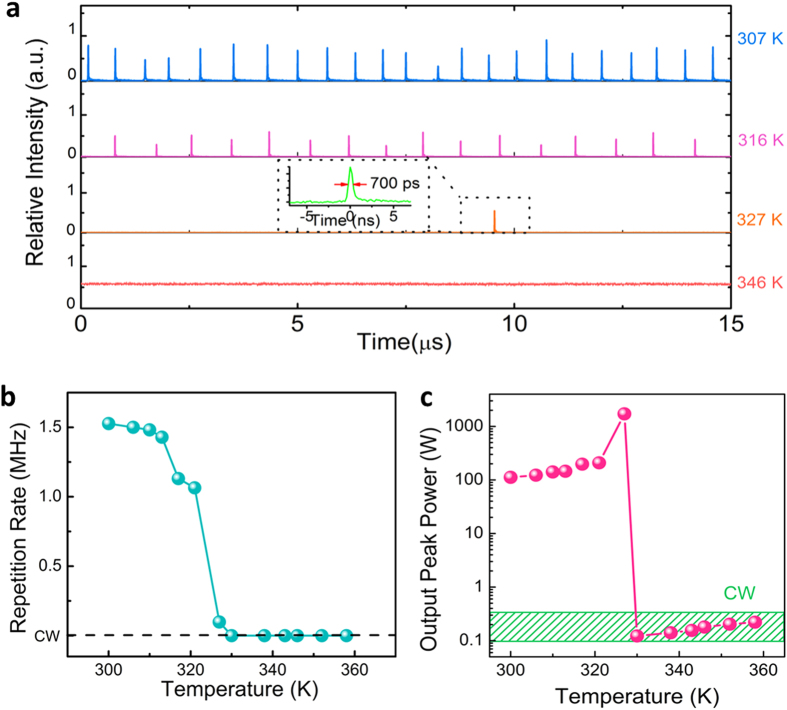
(**a**) Pulse trains of Q-switched waveguide laser at different temperatures. Inset: pulse duration at 327 K. (**b**) repetition rate and (**c**) peak power at different temperatures.

The phase transition of the VO_2_ can also be triggered by increasing the temperature above the threshold (~330K) as shown in Fig. 1c. For the pulsed laser experiments mentioned in the previous sections, it was discovered that when we heat up the VO_2_ sample from its monoclinic phase (307K), the repetition rate of the pulsed laser decreases (Fig. 5a,b). It loses the pulsed laser feature completely when the temperature is higher than 346K. (Details of experiments can be found in the supplementary S3) The variation of the peak power is also shown in Fig. 5c, which is consistent with the variation of the repetition rate. Taking into account of the transmittance of the VO_2_ film, the light power inside the cavity should be more than 200 W. It was proposed in the previous literature that the heating induced phase transition may affect the light induced phase transition^37^. Below the heating phase transition temperature, the heating effect doesn’t trigger the phase transition and the absorption stage is not affected. When the temperature exceeds the phase transition threshold, phase transition cannot happen and the output laser becomes continuous, which is another evidence that the Contrast Nonlinear Transmission is the cause of the observed nonlinearity. It should be noted that the analysis of the detailed dynamics in the recovery stage requires the structural and thermal characterizations in ultra-short time domain. This VO_2_ pulsed waveguide laser setup is the first to demonstrate the direct repetition rate tuning features via temperature. Conventionally, the repetition rate of most of Q-switching waveguide lasers can only be tuned by varying the pump laser power, which greatly affects the output power. To have a laser output with stable and constant power is important for pulsed laser applications. Hence this VO_2_ Contrast Nonlinear Transmission material is promising to be the building block for new photonic devices for the pulsed laser deposition and laser lithography.

## Conclusions

In summary, relying on the phase change materials, an ultrafast and strong nonlinear optical process can be triggered by the laser signal. Based on this mechanism, huge saturable absorption was observed in the experiments. A Q-switching waveguide pulsed laser was fabricated and it demonstrated the shortest pulse duration (700 ps) comparing to other similar types. Furthermore, the analysis on the contrast at different intensities shows that the Contrast Nonlinear Transmission has even greater performance for the devices operation at a higher laser power. This discovery would have general interests to topics related to the nonlinear optical materials. For instance, photonic transistors which rely on ultrafast response and large optical nonlinearity can adapt the functional materials with such mechanism. Moreover, this discovery reveals advantages of the optical nonlinear properties based on the difference of multiple nonlinear phases. In addition, it should be pointed out that the qualitative explanation of the model for Contrast Nonlinear Transmission requires further detailed quantitative investigation to reveal the initial physical mechanism in future work.

## Additional Information

**How to cite this article**: Tan, Y. *et al.* Tunable Picosecond Laser Pulses via the Contrast of Two Reverse Saturable Absorption Phases in a Waveguide Platform. *Sci. Rep.* **6**, 26176; doi: 10.1038/srep26176 (2016).

## Supplementary Material

Supplementary Information
