# Supplementary material for: Tunable Picosecond Laser Pulses via the Contrast of Two Reverse Saturable Absorption Phases in a Waveguide Platform
Source: Sci Rep. 2016 May 18;6:26176. doi: 10.1038/srep26176 (PMC5181842; doi:10.1038/srep26176)
Supplement: Supplementary Information [file srep26176-s1.pdf]

Supplementary Materials

Tunable Picosecond Laser Pulses via the Contrast of Two Reverse Saturable Absorption Phases in a Waveguide Platform

Tan Yang[1]*, CHEN Lianwei[23]*, Dong Wang[1], Yanxue Chen[1], Shavkat Akhmadaliev[3], Shengqiang Zhou[3], Hong Minghui[4] and Chen Feng[1]†

*Equal Contribution

†Contact Author: drfchen@sdu.edu.cn

[1] School of Physics, State Key Laboratory of Crystal Materials, Shandong University, Jinan, China, 250100

[2] NUS Graduate School for Integrative Sciences and Engineering, National University of Singapore, 28 Medical Drive, Singapore, 117456

[3] Helmholtz-Zentrum Dresden-Rossendorf, Institute of Ion Beam and Materials Research, Dresden, Germany, 01314

[4] Department of Electrical and Computer Engineering, National University of Singapore, 4 Engineering Drive 3, Singapore, 117576

S1

The electrical resistance of VO_2 undergoes sharp change across the structural phase transition. Fig. 1s shows the resistance-temperature (R-T) hysteresis curve measured during cooling and heating process at a rate of 1K/min. The inset plot shows the differential of log(R), whose peaks suggest the transition critical temperature, denoted as T_C. The large electrical resistance change ratio (~5×10^4), sharp transition slope and narrow hysteresis window ($\Delta H = T_{C1} - T_{C2}$) indicate good MIT characteristics and hence a good crystal quality of the prepared VO_2 film.

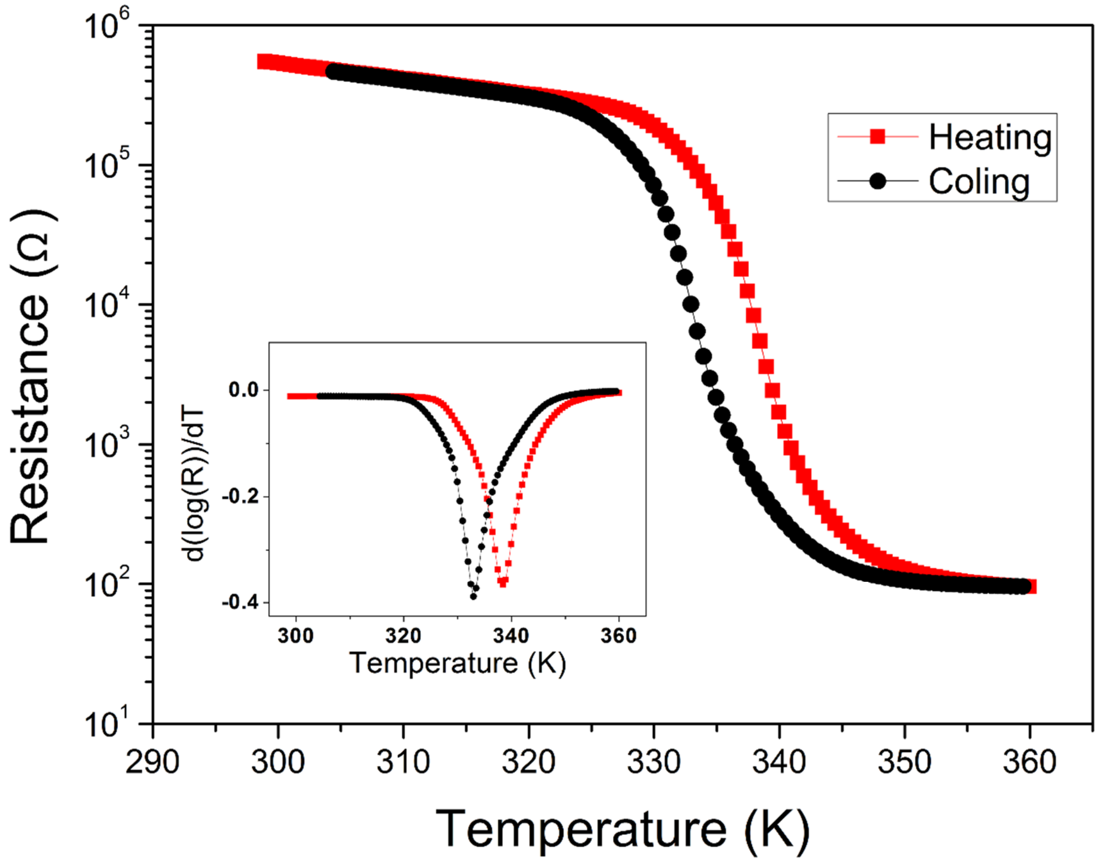

Fig. 1s Resistance-temperature (R-T) hysteresis curve of the prepared VO_2 (110) film. Inset: the differential of log(R).

S2

The saturable intensity of the VO_2 film was fitted according to the modulation depth variation of the pulse waveguide laser. At the room temperature, the modulation depth (ΔT) of the measured pulse laser was calculated by the equation below:

$$\Delta T = \frac{3.52 T_R}{\tau} , \tag{S1}$$

where T_R is the cavity round-trip time and τ the pulse duration. The variation of the modulation depth as a function of the average output power was shown in Fig. 2s.

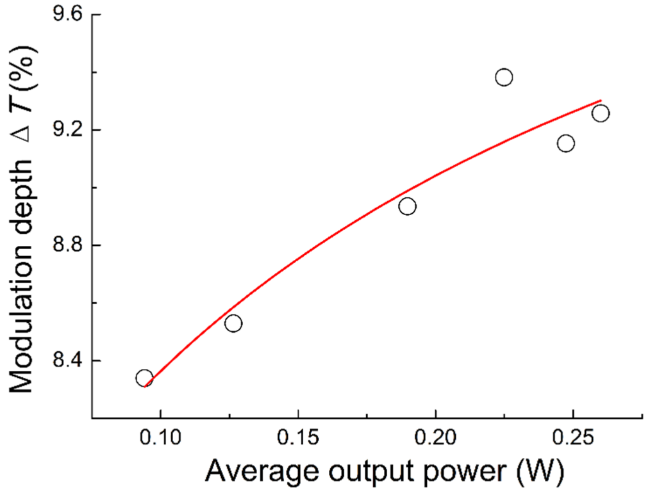

Fig. 2s The variation of the modulation depth as a function of the average output power (black circles) and the fitted line (red solid line).

For the saturable absorption, the modulation depth stands for the difference between the linear and nonlinear transmission of the VO_2 film under the laser irradiation. Hence, the modulation depth (or transmission variation) of the VO_2 film can be fitted by equations below:

$$\alpha(I) = \alpha_{NS} + \frac{\alpha_s}{1 + \frac{I}{I_s}} \quad , \qquad \text{(S2)}$$

$$\Delta T(I) = e^{-\alpha(I)} - T_L \quad , \qquad \text{(S3)}$$

where ΔT is the modulation depth with the irradiation laser power of I, T_L the linear transmission of the VO_2 film, α the absorption coefficient, I_s the saturable intensity, α_s and α_{NS} are saturable and nonsaturable absorption components. The fitted saturable intensity was 89.7 kW/cm^2.

The transmittances of the VO2 film in the insulator and metallic were calculated by equations below: (the results are displayed in Fig. 4b in the manuscript)

$$\alpha = \alpha_L + \beta I \, , \qquad \text{(S4)}$$

$$T = e^{\alpha} \; , \qquad \text{(S5)}$$

where T is the transmission of the VO_2 film, α_L and β the linear and nonlinear absorption coefficients, respectively. α_L and β were directly measured and shown in Figs. 1c and 2. The measured parameters at the insulator and metallic states were substituted into equations, respectively.

S3

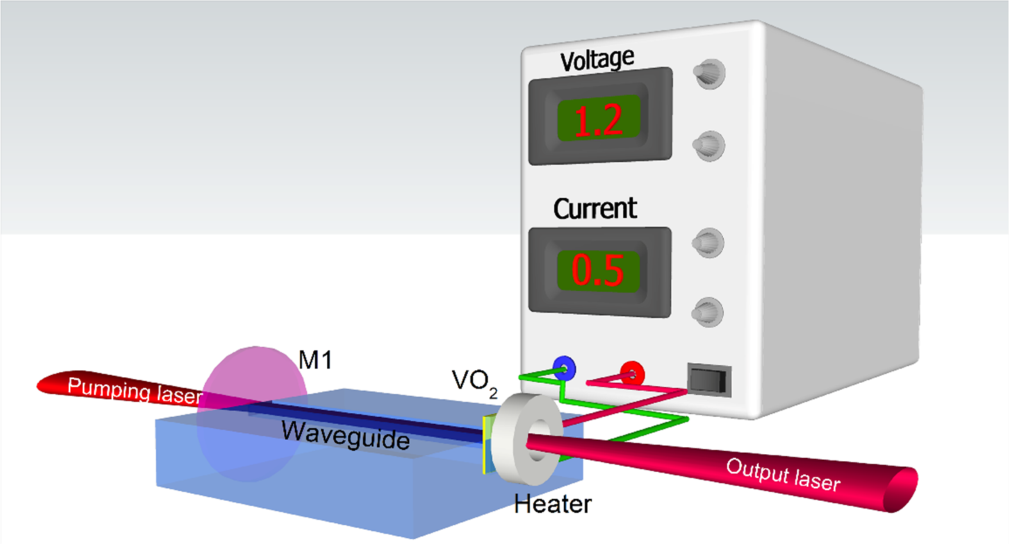

Fig. 3s Experimental setup of the Q-switching waveguide laser.

The waveguide used in this work was fabricated by the swift carbon ion irradiation with the energy of 17 MeV at a fluence of 2×10^{14} Ions/cm^2 in the Nd:YAG ceramic. The Nd:YAG ceramic used in this work was doped by 2 at.% Nd^{3+} ions, obtained from Baikowski Ltd., Japan. During the ion irradiation process, a metal mask with a slit (width of 20 μm and length of 7 mm) was put on the surface of the Nd:YAG ceramic. Under the protection of the mask, the channel waveguide with the width of 20 μm was formed. The propagation loss of the Nd:YAG waveguide was measured to be 0.8 dB/cm at the wavelength of 1064 nm.

The experimental setup for the Q-switched waveguide laser was shown in Fig. 3s. The mirror with the high reflectivity at 1064 nm (>99.9%) and the high transmission at 810 nm (>99.5%) was used as

the input mirror and adhered to the input facet of the Nd:YAG waveguide. The VO_2 film was compressed tightly onto the output facet as the saturable absorber. A cw laser at 810 nm from a Ti:Sapphire laser system was applied as the pump laser. With a lens (focal length of 20 mm), the pump light was coupled into the waveguide passing through the input mirror. The laser oscillation in the waveguide was modulated by the VO_2 film and the output laser from the VO_2 film was collected by a long work distance microscope objective (N.A. = 0.4). The phase of the VO_2 film was controlled by a heating plate. The functions of the heating and cooling were provided by the input voltage.
